# Ascomycetes from the Qilian Mountains, China – Hypocreales

**DOI:** 10.3897/mycokeys.71.55009

**Published:** 2020-08-12

**Authors:** Zhao-Qing Zeng, Huan-Di Zheng, Xin-Cun Wang, Sheng-Long Wei, Wen-Ying Zhuang

**Affiliations:** 1 State Key Laboratory of Mycology, Institute of Microbiology, Chinese Academy of Sciences, Beijing 100101, China Chinese Academy of Sciences Beijing China; 2 Gansu Engineering Laboratory of Application Mycology, Hexi University, Zhangye 734000, China Hexi University Zhangye China

**Keywords:** Biodiversity, Hypocreaceae, Nectriaceae, sequence analysis, taxonomy

## Abstract

To investigate fungi from the Qilian Mountains in Gansu Province, ascomycetous specimens were collected and hypocrealean fungi were examined. Eighteen species belonging to six genera in the families Hypocreaceae and Nectriaceae were identified, including 11 species of *Hypomyces* and *Trichoderma* in Hypocreaceae and seven species of *Nectria*, *Stylonectria*, *Thelonectria*, and *Thyronectria* in Nectriaceae. Among them, *Stylonectria qilianshanensis* and *Trichoderma gansuanum* are new to science. DNA sequence analyses of combined ACL1, ITS, RPB2, and TEF1 regions confirmed their taxonomic placements. Morphological distinctions between the new species and their close relatives are discussed. *Hypomyces tremellicola* is reported for the first time in China.

## Introduction

The Qilian Mountains is located across the northeastern Qinghai and western Gansu provinces (35°50'–39°19'N, 94°10'–103°04'E) at elevations ranging from 4000 to 6000 m. The area has a temperate climate with mean annual precipitation around 400 mm. Forests are composed mainly of mixed broad-leaf and coniferous trees. The Qilian Mountain National Nature Reserve, where ascomycetes were surveyed, is extremely diverse in climate, vegetation, and geographic structure. Previous investigations of fungal resources have mainly focused on Basidiomycota (Gui et al. 2010; Xi et al. 2011). Our understanding of ascomycetes of the region needs to be broadened.

The order Hypocreales includes about 2700 species in 240 genera, which are divided into 12 families (Rossman et al. 1999; Lumbsch and Huhndorf 2007; Kirk et al. 2008; Lombard et al. 2015; Maharachchikumbura et al. 2016; Sun et al. 2017; Zhuang and Zeng 2017). Hypocrealean collections from the Qilian Mountains belong to the families Hypocreaceae and Nectriaceae. These families are ubiquitous in nature and exhibit very high species diversity in temperate and tropical regions (Rossman 1996; Rossman et al. 1999). They are economically important in fields of industry, environment protection, and agriculture. For instance, some species of *Trichoderma* Pers. play vital roles in production of industrial enzymes and antibiotics (Jangiret et al. 2017), while several species of *Hypomyces* (Fr.) Tul. & C. Tul. are pathogens of cultivated mushrooms (Tamm and Põldmaa 2013). Some members of *Nectria* (Fr.) Fr., *Thelonectria* P. Chaverri & C. Salgado, and *Thyronectria* Sacc. cause *Abies* and *Rubus* cankers (Hirooka et al. 2011, 2012; Salgado-Salazar et al. 2015). Therefore, discovery of fungi in Hypocreales is of theoretical and practical importance. Improvement and updating our knowledge of the group will provide useful information about sustainable utilization and conservation of natural resources.

Studies on fungi of this group in China dates back to 1895 when *Trichoderma cornu-damae* (Pat.) Z.X. Zhu & W.Y. Zhuang (as *Hypocrea cornu-damae* Pat.) was reported on rotten wood in Sichuan Province (Patouillard 1895). Early studies were initiated by Teng (1934, 1935, 1936, 1963), and recent studies are summarized by Liang (2007), Zhuang (2013), and Zhuang and Zeng (2017). A survey of ascomycetes in the Qilian Mountains was carried out in 2018. A total of 67 specimens were examined in this study. Eighteen taxa belonging to seven genera were identified, including 11 species of *Hypomyces* and *Trichoderma* in Hypocreaceae, and seven of *Nectria*, *Thelonectria*, *Thyronectria*, and *Stylonectria* Höhn. in Nectriaceae. *Stylonectria qilianshanensis* and *Trichoderma gansuanum* are described and illustrated as new species. *Hypomyces tremellicola* is reported for the first time from China.

## Materials and methods

### Sampling and morphological studies

Specimens were collected from the Qilian Mountains in Gansu Province, and they are deposited in the Herbarium Mycologicum Academia Sinica (HMAS). Cultures are kept in the State Key Laboratory of Mycology, Institute of Microbiology, Chinese Academy of Sciences. The methods of Luo and Zhuang (2010) and Põldmaa et al. (2019) were followed for morphological observations. The ascomatal wall reactions to 3% potassium hydroxide (KOH) and 100% lactic acid (LA) were tested. To observe microscopic characteristics of perithecial walls, sections were made with a freezing microtome (YD-1508-III, Jinhua, China) at a thickness of 6–8 μm. Cotton blue lactophenol solution and lactic acid solution were used as mounting media for examinations of anatomical structures and measurements of perithecia, asci, and ascospores. Photographs were taken with a Leica DFC450 digital camera (Wetzlar, Germany) attached to a Leica M125 stereomicroscope (Milton Keynes, UK) for gross morphology, and a Zeiss AxioCamMRc 5 digital camera (Jena, Germany) attached to a Zeiss Axio Imager A2 microscope (Göttingen, Germany) for anatomy. Measurements of individual structures were based on *n* = 30, except as otherwise noted. The culture of *Hypomyces tremellicola* was isolated from conidia found on the surface of the host. To determine colony features and growth rates, strains were grown on malt extract agar [MEA, 2% (w/v) malt extract+ 2% (w/v) agar] and potato dextrose agar [PDA, 20% (w/v) potato + 2% (w/v) dextrose + 2% (w/v) agar] in 90 mm plastic Petri dishes at 25 °C for 7 d. For observation of conidiophores and conidia, cultures were grown on PDA at 25 °C with alternating periods of light and darkness (12 h/12 h).

### DNA extraction, PCR amplification, and sequencing

Genomic DNA was extracted from dry specimens or fresh mycelia following the method of Wang and Zhuang (2004). Four primer pairs, acl1-230up/acl1-1220low (Nowrousian et al. 2000), ITS5/ITS4 (White et al. 1990), RPB2-5f/RPB2-7cR (Liu et al. 1999), EF1-728F/TEF1LLErev (Carbone and Kohn 1999; Jaklitsch et al. 2005) were used to amplify the ACL1, ITS, RPB2, and TEF1 gene regions, respectively. PCR reactions were performed using an ABI 2720 Thermal Cycler (Applied Biosciences, Foster City, USA) with a 25 μl reaction system consisting of 12.5 μl Taq MasterMix, 1 μl each primer (10 μM), 1 μl template DNA, and 9.5 μl ddH_2_O. DNA sequencing was carried out in both directions on an ABI 3730XL DNA Sequencer (Applied Biosciences, Foster City, USA) based on the procedures detailed in Gräfenhan et al. (2011), Jaklitsch et al. (2005), and Chaverri et al. (2011).

### Sequence alignment and phylogenetic analyses

Newly obtained sequences and those retrieved from GenBank are listed in Tables 1 and 2, respectively. The sequences were assembled, aligned and the primer sequences were trimmed using BioEdit 7.0.5 (Hall 1999), and converted to NEXUS files by ClustalX 1.83 (Thompson et al. 1997). The aligned sequences were combined in BioEdit and analyzed with Bayesian inference (BI) and maximum parsimony (MP) methods to determine the phylogenetic positions of the new species. The MP analysis was performed with PAUP 4.0b10 (Swofford 2002) using 1000 replicates of heuristic search with random addition of sequences and subsequent TBR (tree bisection and reconnection) branch swapping. Topological confidence of the resulting trees was tested by maximum parsimony bootstrap proportion (BP) with 1000 replications, each with 10 replicates of random addition of taxa. The Bayesian inference (BI) analysis was conducted by MrBayes 3.1.2 (Ronquist and Huelsenbeck 2003) using a Markov chain Monte Carlo algorithm. MrModeltest v. 2.3 was used to determine the nucleotide substitution models (Nylander 2004). Four Markov chains were run simultaneously for 1000000 generations with the trees sampled every 100 generations. A 50% majority rule consensus tree was computed after excluding the first 2500 trees as ‘burn-in’. Bayesian inference posterior probability (PP) was determined from the remaining trees. Branch support measures were calculated with 1000 bootstrap replicates. Trees were examined by TreeView 1.6.6 (Page 1996). The BIPP greater than 90% and MPBP greater than 70% were shown at the nodes.

**Table 1.** List of *Stylonectria* species and the relatives, herbarium/strain numbers and GenBank accession numbers of materials used in this study.

Species	Herbarium/strain numbers	GenBank accession numbers		
		ACL1	ITS	RPB2
*Albonectri rigidiuscula* (Berk. & Broome) Rossman & Samuels	CBS 122570	HQ897896	HQ897815	HQ897760
*Clonostachys rosea* (Preuss) Mussat	CML817/CBS 114056	KX184866	KC806254	DQ522415
*Cyanonectria cyanostoma* (Sacc. & Flageolet) Samuels & P. Chaverri	CBS 101734	HQ897895	FJ474076	HQ897759
*Dialonectria episphaeria* (Tode) Cooke	CBS 125494	HQ897892	HQ897811	HQ897756
*Fusarium sambucinum* Fuckel	CBS 14695	KM231015	KM231813	KM232381
*Fusicolla matuoi* (Hosoya & Tubaki) Gräfenhan & Seifert	CBS 58178	HQ897858	KM231822	HQ897720
*Geejayessia cicatricum* (Berk.) Schroers	CBS 125552	HQ728171	HQ728145	HQ728153
*Macroconia leptosphaeriae* (Niessl) Gräfenhan & Schroers	CBS 100001	HQ897891	HQ897810	HQ897755
*Microcera coccophila* Desm.	CBS 31034	HQ897843	HQ897794	HQ897705
*Neocosmospora vasinfecta* E.F. Sm.	CBS 32554	KM231005	KM231803	KM232370
*Stylonectria applanata* Höhn.	CBS 125489	HQ897875	HQ897805	HQ897739
*Stylonectria carpini* Gräfenhan	DAOM 235819	HQ897909	HQ897823	HQ897773
*Stylonectria norvegica* Lechat, J. Fourn. & Nordén	CBS 139239	–	NR154415	–
*Stylonectria purtonii* (Grev.) Gräfenhan	DAOM 235818	HQ897919	HQ897831	HQ897783
*Stylonectria qilianshanensis* Z.Q. Zeng & W.Y. Zhuang	HMAS 255803	**MT087289** ^a^	**MT084413**	**MT087288**
*Stylonectria wegeliniana* (Rehm) Gräfenhan, Voglmayr & Jaklitsch	CBS 125490	HQ897890	KM231817	HQ897754
*Trichoderma parareesei* Atan., Jaklitsch, Komoń-Zel., C.P. Kubicek & Druzhin.	CBS 125925	KJ665112	MH863773	HM182963

^a^ Numbers in bold indicate the newly provided sequences.

**Table 2.** List of *Trichoderma* species, herbarium/strain numbers, and GenBank accession numbers of specimens used in this study.

Species	Herbarium/strain numbers	GenBank accession numbers	
		RPB2	TEF1
*Hypocrella discoidea* (Berk. & Broome) Sacc.	BCC 8237	DQ452461	–
*Hypocrella nectrioides* Thaxt.	GJS 8910	DQ522448	–
*Trichoderma alutaceum* Jaklitsch	CBS 120535	FJ179600	FJ179567
	CBS 33269	FJ179601	FJ179568
*Trichoderma gansuanum* Z.Q. Zeng & W.Y. Zhuang	HMAS 279687	**MT087287** ^a^	**MT095060**
*Trichoderma gelatinosum* P. Chaverri & Samuels	CPK 1618	FJ179604	FJ179569
*Trichoderma leucopus* Jaklitsch	CBS 122495	FJ179606	FJ179570
	CBS 122499	FJ179605	FJ179571
*Trichoderma lixii* (Pat.) P. Chaverri	CPK 1934	FJ179608	FJ179573
*Trichoderma minutisporum* Bissett	CBS 121276	FJ179610	FJ179574
*Trichoderma nybergianum* (T. Ulvinen & H.L. Chamb.) Jaklitsch & Voglmayr	CBS 122496	FJ179612	FJ179576
	CBS 122500	FJ179611	FJ179575
*Trichoderma parapiluliferum* (B.S. Lu, Druzhin. & Samuels) Jaklitsch & Voglmayr	CBS 20921	FJ179614	FJ179578
*Trichoderma pezizoides* (Berk. & Broome) Samuels, Jaklitsch & Voglmayr	GJS 01257	EU248608	AY937438
*Trichoderma piluliferum* J. Webster & Rifai	CBS 120927	KJ842159	FJ860674
*Trichoderma placentula* Jaklitsch	CBS 120924	FJ179616	FJ179580
*Trichoderma polysporum* (Link) Rifai	CPK 3131	FJ860558	FJ860661
*Trichoderma poronioideum* (Möller) Samuels	GJS 01203	–	KP109823
*Trichoderma seppoi* Jaklitsch	CBS 122497	FJ179618	FJ179582
	CBS 122498	FJ179617	FJ179581
*Trichoderma strictipile* Bissett	CPK 1601	FJ860594	FJ860704

^a^ Numbers in bold indicate the newly provided sequences.

## Results

To determine taxonomic positions of the *Hypomyces* collections, sequences of ITS and 28S rDNA were searched against the NCBI GenBank database using BLASTN. Sequence comparisons showed that HMAS 247843 shares 99% sequence similarity with *H. tremellicola*, which confirmed its taxonomic position in the genus.

**Figure 1.**
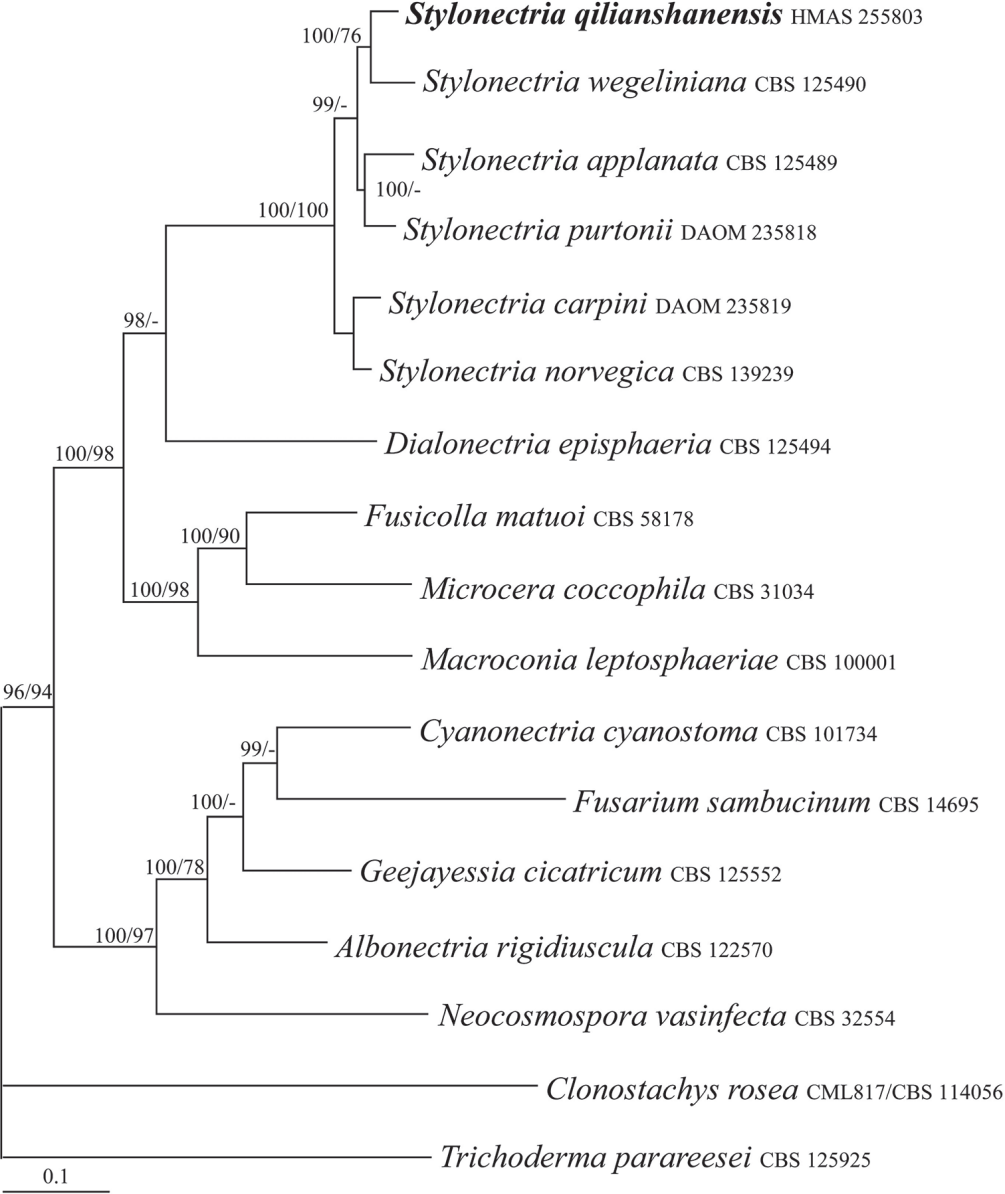
BI tree generated based on the combined datasets of ACL1, ITS and RPB2 sequences of *Stylonectria* and its relatives. Supporting values showing at branches: BIPP (left) and MPBP (right). BIPP greater than 90% and MPBP greater than 70% are shown at the nodes.

To place the *Stylonectria* specime n within a phylogenetic context, sequences of ACL1, ITS, and RPB2 regions from 15 species of the genus and relatives were analyzed using BI and MP methods. *Clonostachys rosea* (Preuss) Mussat and *Trichoderma parareesei* Atan., Jaklitsch, Komoń-Zel., C.P. Kubicek & Druzhin. were used as outgroup taxa. The partition homogeneity test (PHT) (*P* = 0.01) indicated that the individual partitions were not highly incongruent (Cunningham 1997), the three loci were thus combined for phylogenetic analyses. The combined datasets include 2197 characters, of which 964 were constant, 310 were variable and parsimony-uninformative and 923 were parsimony-informative. The MP analysis resulted in two most parsimonious trees (tree length = 4081, CI = 0.5285, HI = 0.4715, RI = 0.4459, RCI = 0.2357) with similar topology. The final matrix was deposited in TreeBASE with accession no. S25189. The BI tree is shown in Figure 1. The MP tree is similar to that of the BI tree in topology. HMAS 255803 was associated with other *Stylonectria* species forming a highly supported monophyletic group (BIPP/MPBP = 100%/100%), which confirmed its taxonomic position in the genus.

**Figure 2.**
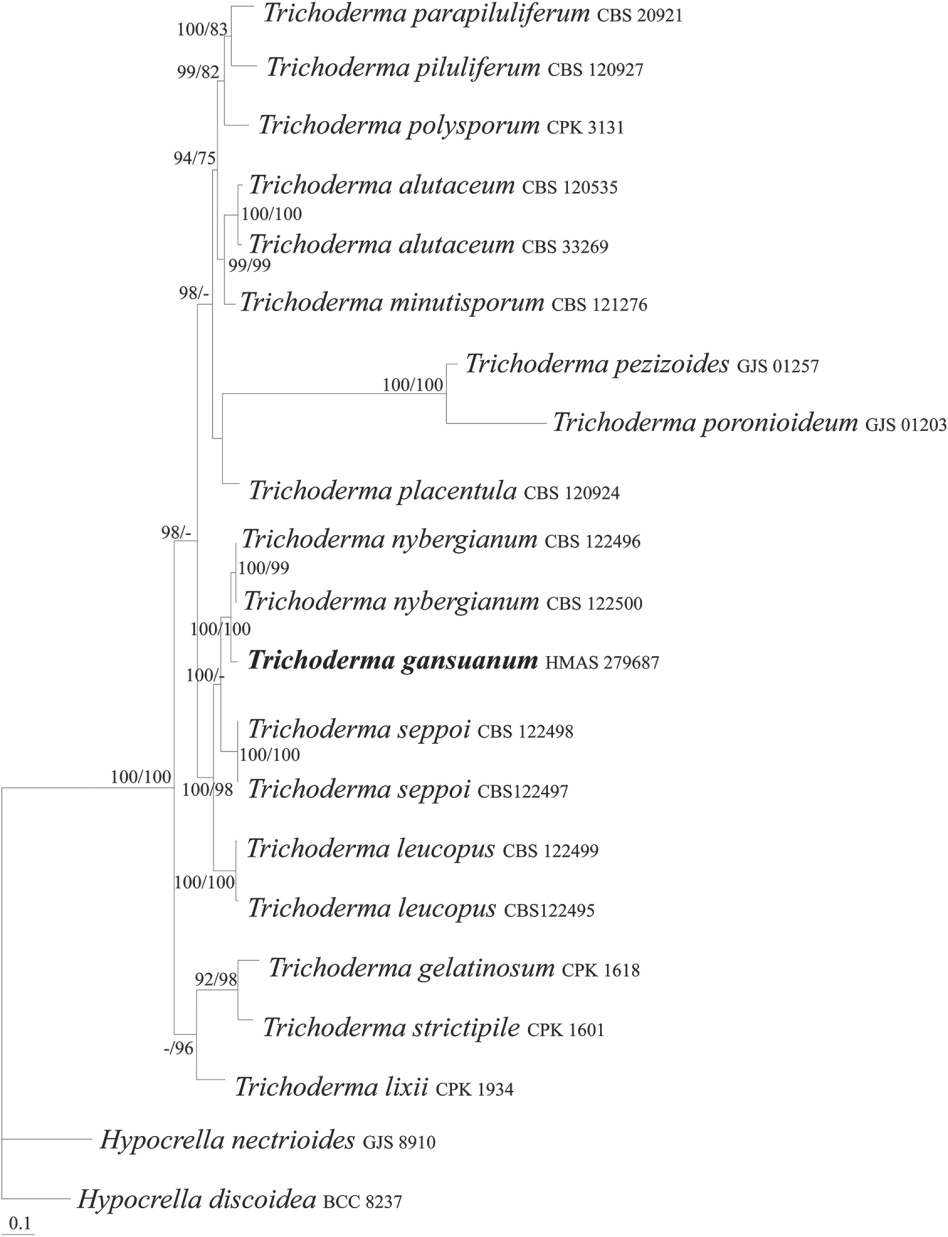
BI tree generated based on the combined datasets of RPB2 and TEF1 sequences of *Trichoderma* species. Supporting values showing at branches: BIPP (left) and MPBP (right). BIPP greater than 90% and MPBP greater than 70% are shown at the nodes.

To place the *Trichoderma* collections with clavate fruit bodies within a phylogenetic context, the sequences of RPB2 and TEF1 from 15 species of the genus were analyzed using BI and MP. *Hypocrella discoidea* (Berk. & Broome) Sacc. and *Hypocrella nectrioides* Thaxt. were used as outgroup taxa. The PHT (*P* = 0.01) indicated that the individual partitions were not highly incongruent (Cunningham 1997), the two loci were thus combined for phylogenetic analyses. The combined datasets include 1436 characters, of which 688 were constant, 167 were variable and parsimony-uninformative and 581 were parsimony-informative. The MP analysis resulted in one most parsimonious tree (tree length =1828, CI = 0.6510, HI = 0.3490, RI = 0.6706, RCI = 0.4366). The final matrix was deposited in TreeBASE with accession no. S25188. The BI tree is shown in Figure 2, which is similar to the MP tree in topology. Among the investigated species, HMAS 279687 was distinct from but associated with other *Trichoderma* species forming a highly supported monophyletic group (BIPP/MPBP = 100%/100%), which confirmed its taxonomic position.

### Taxonomy

#### Stylonectria qilianshanensis

Taxon classificationFungiHypocrealesNectriaceae

Z.Q. Zeng & W.Y. Zhuang sp. nov.

AB84A707-F954-538F-B76C-D0ED2EB9FA61

Fungal Names: FN 570729

Figure 3

##### Holotype.

China. Gansu Province, Wuwei, Chashugou, on decayed fruitbodies of an ascomycete on bark of *Picea asperata*, 26 August 2018, Z.Q. Zeng, X.C. Wang & H.D. Zheng 12155 (HMAS 255803).

##### Paratypes.

China. Zhangye, Longchanghe, on decayed fruitbodies of an ascomycete on bark of *Picea asperata*, 24 August 2018, Z.Q. Zeng, X.C. Wang & H.D. Zheng 12016, 12017 (HMAS 255804, 255805); Kangle, on decayed fruitbodies of an ascomycete on bark of *Picea asperata*, 24 August 2018, Z.Q. Zeng, X.C. Wang & H.D. Zheng 12035, 12036, 12037, 12038 (HMAS 255806, 255807, 255808, 255809); Shandan, Yanzhishan, on decayed fruitbodies of an ascomycete on bark of *Picea asperata*, 25 August 2018, H.D. Zheng, X.C. Wang & Z.Q. Zeng 12082 (HMAS 255810); Yanzhishan, on decayed fruitbodies of an ascomycete on bark of *Picea asperata*, 25 August 2018, Z.Q. Zeng, X.C. Wang & H.D. Zheng 12086, 12087, 12088, 12089, 12090 (HMAS 255811, 255812, 255813, 255814, 255815); Wuwei, Chashugou, on decayed fruitbodies of an ascomycete on bark of *Picea asperata*, 26 August 2018, Z.Q. Zeng, X.C. Wang & H.D. Zheng 12148, 12153, 12156, 12158 (HMAS 255816, 255817, 255818, 279708); Tianzhu, Kelacun, on decayed fruitbodies of an ascomycete on bark of *Picea asperata*, 27 August 2018, Z.Q. Zeng, X.C. Wang & H.D. Zheng 12229 (HMAS 279709); Haxi, on decayed fruitbodies of an ascomycete on bark of *Picea asperata*, 28 August 2018, Z.Q. Zeng, X.C. Wang & H.D. Zheng 12276, 12278 (HMAS 255819, 255820).

##### Etymology.

The specific epithet refers to the type locality.

##### Description.

Perithecia gregarious, up to 30 in a group, parasitic on decayed fruitbodies of an ascomycete, deep red to dark red, turning black red in 3% KOH and light yellow in 100% LA, subglobose to globose, not becoming cupulate upon drying, (216–)255–344 × (186–)206–304 μm (*n* = 12), apex broadly discoid, flattened, 50–70 μm high, 160–220 μm in diameter, slightly constricted below, with a tiny papilla. Perithecial wall of two layers, 25–38 μm thick, outer layer 20–31 μm, of textura angularis, cells 5–8 × 2–4 μm, walls 0.8–1.0 μm thick; inner layer 5–7 μm, of textura prismatica, cells 8–12.5 × 3–5 μm, walls 0.5–0.8 μm thick. Asci clavate, with an apical ring, 8-spored, 55–88 × 5–8(–10) μm. Ascospores ellipsoidal, ends rounded, 1-septate, light brown, smooth, uniseriate, 10–13 × 5–5.5 μm. Asexual state unknown.

##### Distribution.

China.

##### Notes.

*Stylonectria qilianshanensis* is morphologically similar to *S. wegeliniana* in having the perithecia with a broad, discoid apex, clavate asci with an apical ring, and ellipsoidal ascospores with rounded ends (Petch 1938). However, *S. qilianshanensis* differs in having smaller asci [55–88 × 5–8(–10) μm vs 90–100 × 9–10 μm] and ascospores (10–13 × 5–5.5 μm vs 10–18 × 6–9 μm) (Petch 1938). Sequence comparisons indicate that ITS of *S. qilianshanensis* differs from that of *S. wegeliniana* by 20 bp in a total length of 576 bp; ACL1 and RPB2 of the former differ from those of the latter by 84 bp and 38 bp, respectively among 722 bp and of 869 bp in length. The asexual state of the fungus remains unknown until culture is available.

**Figure 3.**
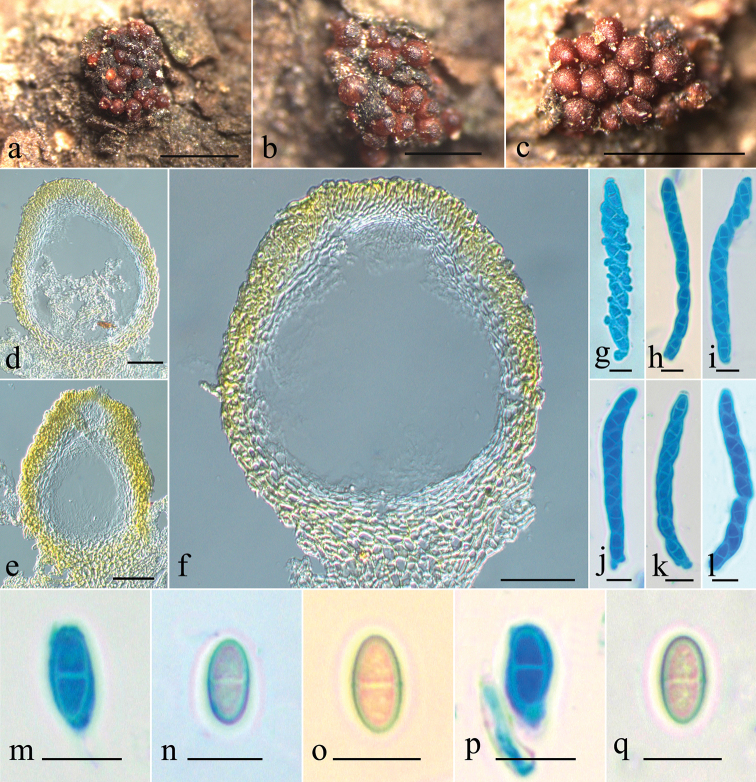
*Stylonectria qilianshanensis***a–c** ascomata on natural substrate **d–f** median section through perithecium **g–l** ascus with ascospores **m–q** ascospores. From HMAS 255803. Scale bars: 1 mm (**a–c**); 50 μm (**d–f**); 10 μm (**g–q**).

#### Trichoderma gansuanum

Taxon classificationFungiHypocrealesHypocreaceae

Z.Q. Zeng & W.Y. Zhuang sp. nov.

A99012CE-1114-5413-8E7C-8387E26A8B77

Fungal Names: FN 570730

Figure 4

##### Holotype.

China. Gansu Province, Shandan, Yanzhishan, on mossy humus, 25 August 2018, Z.Q. Zeng, H.D. Zheng & X.C. Wang 12100 (HMAS 279687).

##### Paratypes.

China. Gansu Province, Shandan, Yanzhishan, on mossy humus, 25 August 2018, Z.Q. Zeng, H.D. Zheng 12043, 12044 (HMAS 279684, 279685), on mossy humus, 25 August 2018, X.Z. Liu & Z.Q. Zeng 12045 (HMAS 279686); Wuwei, Chashugou, on mossy humus, 26 August 2018, Z.Q. Zeng, H.D. Zheng & X.C. Wang 12104, 12105 (HMAS 279688, 279689); Wuwei, Xiama, on mossy humus, 26 August 2018, Z.Q. Zeng, H.D. Zheng & X.C. Wang 12162, 12163 (HMAS 279690, 279691).

##### Etymology.

The specific epithet refers to the type locality.

##### Description.

Stromata simple, rare dichotomously branched, clavate, 20–54 mm long. Fertile part clavate, 5–18 mm long, 1.2–4 mm wide at apex, only slightly broader than stipe, distinctly laterally compressed or longitudinally furrowed, gradually tapered downwards, reddish brown to brownish orange, KOH+; sterile part 15–36 mm long, 1–3 mm wide, beige to cream KOH+. Stromatal surface slight tuberculate from papillate perithecial elevations. Ostiolar openings visible, 30–58 μm high. In section, cortical tissue of textura angularis,15–35 μm thick, cells hyaline to light yellow, 5–15 × 2–3 μm; subcortical tissue of textura angularis, 8–28 μm thick, cell hyaline to light yellow, 8–15 × 3–5 μm; subperithecial tissue of textura epidermoidea, rare textura angularis, cells hyaline to light yellow. Perithecia globose to subglobose, 196–206 × 167–235 μm; peridium 8–18 μm thick at flanks, 9–20 μm thick at the base. Papilla prominent, blunt or truncate, brown, 15–35 μm high, 18–43 μm wide at the base. Asci subcylindrical, 50–80 × 3–4 μm. Part-ascospores green, spinulose, dimorphic, distal cells globose, rarely ellipsoidal, 2.5–4 × 2.5–4 μm; proximal cells ellipsoidal to oblong, 3–5.5 × 2.5–3 μm. Asexual state unknown.

**Figure 4.**
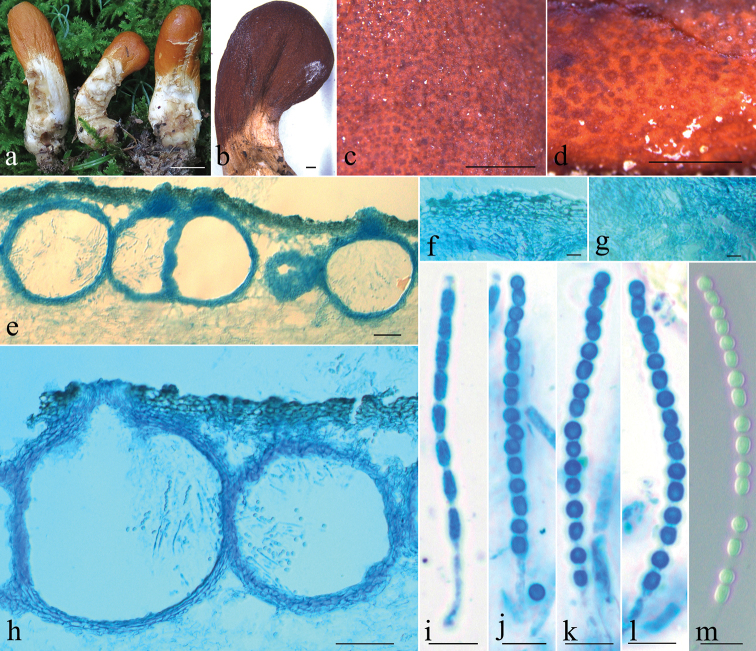
*Trichoderma gansuanum***a** fresh stroma **b** dry stroma **c** stromatal surface **d** color of stroma after rehydration **e** median section through stromata **f** cortical tissue in section **g** subperithecial tissues in section **h** perithecia in section **i** ascus with ascospores **j–m** ascus with part-ascospores **a** from HMAS 279684, **b–m** from HMAS 279687. Scale bars: 1 cm (**a**); 1 mm (**b–d**); 50 μm (**e–f**); 10 μm (**g–m**).

##### Distribution.

China.

##### Notes.

Among the known stipitate species of *Trichoderma*, *T. gansuanum* resembles *T. nybergianum* in habitat and having simple, clavate, erect stromata, cylindrical asci and disarticulating ascospores (Chamberlain et al. 2004). *Trichoderma gansuanum* differs from the latter in shorter ascomatal stipe (20–54 mm vs 22–220 mm long), smaller perithecia (196–206 × 167–235 μm vs 180–450 × 65–315 µm), asci (50–80 × 3–4 μm vs 63–130 × 3.2–7.5 μm), and part-ascospores (distal: 2.5–4 × 2.5–4 μm vs 3–6 × 3–4.5 μm; proximal: 3–5.5 × 2.5–3 μm vs 3–6 × 2.5–4.5 μm) (Chamberlain et al. 2004). Sequence comparisons reveal that there are 25 bp and 16 bp divergences between the two species in the regions of RPB2 and TEF1. Both morphological and molecular data support distinguish them at the species level. The asexual state of *T. gansuanum* remains unknown until a culture is available.

#### Hypomyces cervinigenus

Taxon classificationFungiHypocrealesHypocreaceae

Rogerson & Simms, Mycologia 63: 418, 1971.

FF2337BA-E5CC-5EB5-B592-E1503601E4BB

##### Specimens examined.

China, Gansu Province, Wuwei, Tianzhu, on *Helvella* sp., 26 August 2018, X.C. Wang, Z.Q. Zeng & H.D. Zheng 12103 (HMAS 279612); Wuwei, Tianzhu, on *Helvella* sp., 27 August 2018, Z.Q. Zeng, H.D. Zheng & X.C. Wang 12210, 12221, 12225 (HMAS 279667, 279668, 279669).

##### Distribution.

Canada, China, United States.

#### Hypomyces chrysospermus

Taxon classificationFungiHypocrealesHypocreaceae

Tul. & C. Tul., Annls Sci. Nat., Bot., Sér. 4, 13: 16, 1860.

F1117056-6B2C-59BC-ABFD-B4947C384F52

##### Specimens examined.

China, Gansu Province, Tianzhu, Zhuchacun, on *Boletus* sp., 27 August 2018, Z.Q. Zeng, H.D. Zheng & X.C. Wang 12206 (HMAS 279670); Tianzhu, Kelacun, on *Boletus* sp., 27 August 2018, Z.Q. Zeng, H.D. Zheng & X.C. Wang 12220 (HMAS 279671).

##### Distribution.

Australia, Belgium, Canada, China, French, Japan, New Zealand, United Kingdom, United States.

#### Hypomyces lateritius

Taxon classificationFungiHypocrealesHypocreaceae

(Fr.) Tul. & C. Tul., Annls Sci. Nat., Bot., Sér. 4, 13: 11, 1860.

BE05ED74-6C8B-5D7B-9D1F-30F8AB18CF6F

##### Specimens examined.

China, Gansu Province, Shandan, Yanzhishan, on *Lactarius* sp., 25 August 2018, Z.Q. Zeng, H.D. Zheng & X.C. Wang 12060, 12061 (HMAS 254608, 254609); Tianzhu, Kelacun, on *Lactarius* sp., 27 August 2018, Z.Q. Zeng, H.D. Zheng & X.C. Wang 12216, 12217, 12218, 12219 (HMAS 279672, 279673, 279674, 279675).

##### Distribution.

Austria, Belgium, Canada, China, Czech, Denmark, Finland, France, Germany, Italy, Mexico, New Zealand, Russia, Sweden, United Kingdom, United States.

#### Hypomyces perniciosus

Taxon classificationFungiHypocrealesHypocreaceae

Magnus, Bot. Ztg. 46: 394, 1888.

DC507CBE-27F8-5993-9A44-C988D5D77D53

##### Specimen examined.

China, Gansu Province, Tianzhu, Kelacun, on *Agaricus* sp., 27 August 2018, Z.Q. Zeng, H.D. Zheng & X.C. Wang 12222 (HMAS 279705).

##### Sequences.

ITS (MT676396) and LSU (MT669266).

##### Distribution.

China, France, Germany, United Kingdom.

#### Hypomyces rosellus

Taxon classificationFungiHypocrealesHypocreaceae

(Alb. & Schwein.) Tul. & C. Tul., Annls Sci. Nat., Bot., Sér. 4 13: 12, 1860.

C4B88525-74E2-5726-88D3-61CEEF6B05E2

##### Specimen examined.

China, Gansu Province, Shandan, Yanzhishan, on *Lactarius* sp., 25 August 2018, Z.Q. Zeng, H.D. Zheng & X.C. Wang 12056 (HMAS 279706).

##### Sequences.

ITS (MT676395).

##### Distribution.

China, Estonia, Poland, Ukraine, United States.

#### Hypomyces stephanomatis

Taxon classificationFungiHypocrealesHypocreaceae

Rogerson & Samuels, Mycologia 77: 775, 1985.

7F1C1094-1892-5148-BB02-065FF497C0B0

##### Specimens examined.

China, Gansu Province, Shandan, Yanzhishan, on *Humaria* sp., 25 August 2018, Z.Q. Zeng, H.D. Zheng & X.C. Wang 12063 (HMAS 279676); Tianzhu, Zhuchacun, on *Humaria* sp., 27 August 2018, Z.Q. Zeng, H.D. Zheng & X.C. Wang 12205, 12207, 12208, 12209, 12211 (HMAS 279677, 279678, 279679, 279680, 279681); Tianzhu, Kelacun, on *Humaria* sp., 27 August 2018, Z.Q. Zeng, H.D. Zheng & X.C. Wang 12223, 12226 (HMAS 279682, 279683).

##### Distribution.

Canada, China, Germany, United States.

#### Hypomyces tremellicola

Taxon classificationFungiHypocrealesHypocreaceae

(Ellis & Everh.) Rogerson, Mem. N. Y. Bot. Gdn. 26(3): 20, 1976.

5FA3B33D-75CB-52C6-96DD-567DED3BBECF

##### Specimen examined.

China, Gansu Province, Zhangye, Minyue, on (?) *Agaricus* sp., August 2018, C.H. Dong & S.J. Li 12287 (HMAS 247843).

##### Sequences.

ITS (MT084414) and LSU (MT078664).

##### Description.

On MEA, colony radius 33 mm after 7d at 25 °C, velvet, surface white, reverse light brown; aerial hyphae white. On PDA, colony radius 20 mm after 7d at 25 °C, floccose, surface grey white, reverse light sienna; aerial hyphae white. Simple branches of aerial hyphae terminating in 1–2-verticillate conidiophores with terminal whorl of 2–4 phialides. Phialides subulate, tapering towards apex, smooth, 8–25 × 1.5–2 μm. Conidia ellipsoidal to rod-shaped, aseptate, hyaline, smooth, 2.5–8 × 1–3 μm. Chlamydospores globose, hyaline, smooth, 5–8 μm in diameter, rare ellipsoidal, 6–12 × 5–10 μm, formed singly or in chains in intercalary position.

##### Distribution.

Canada, China, Germany, New Zealand, The Netherlands, United States, Venezuela.

##### Notes.

*Hypomyces tremellicola* is a new record for China. This species was originally described as *Hypocrea tremellicola* Ellis & Everh. (Ellis and Everhart 1892), and later transferred to *Hypocreopsis* P. Karst. (Seaver 1910) and *Nectriopsis* Maire (Gams and Zaayen 1982). Samuels (1976) redescribed the species and assigned it to *Hypomyces*. It usually grows on *Crepidotus* spp., and less frequently on *Polyporus* spp. and *Pleurotus* spp. The shape and size of conidia and chlamydospores of the Chinese material match well with the description provided by Zare and Gams (2019). Sequence comparisons showed that 4 bp and 1 bp divergences existed in ITS and 28S rDNA between the Chinese material (HMAS 247843) and a collection from Germany (CBS 441.65). We treat them as infraspecific variations.

#### Nectria asiatica

Taxon classificationFungiValvatidaGoniasteridae

Hirooka, Rossman & P. Chaverri, Stud. Mycol. 68: 44, 2011.

6C814D34-0870-5F9B-A276-9175C451D96E

##### Specimen examined.

China, Gansu Province, Tianzhu, Zhuchacun, on rotten twigs, 27 August 2018, Z.Q. Zeng, H.D. Zheng & X.C. Wang 12214 (HMAS 254610).

##### Distribution.

China, Japan.

#### Nectria berberidicola

Taxon classificationFungiValvatidaGoniasteridae

Hirooka, Lechat, Rossman & P. Chaverri, in Hirooka, Rossman, Samuels, Lechat & Chaverri, Stud. Mycol. 71: 48, 2012.

D01522AE-09AF-5847-8CD7-C248DA4892E8

##### Specimens examined.

China, Gansu Province, Shandan, Yanzhishan, on rotten twigs, 25 August 2018, Z.Q. Zeng, H.D. Zheng & X.C. Wang 12084 (HMAS 279707); Tianzhu, Zhuchacun, on rotten twigs of *Berberis* sp., 27 August 2018, Z.Q. Zeng, H.D. Zheng & X.C. Wang 12212 (HMAS 254611); Tianzhu, Haxia, on rotten twigs of *Berberis* sp., 28 August 2018, Z.Q. Zeng, H.D. Zheng & X.C. Wang 12279, 12280, 12281 (HMAS 254612, 254613, 255801).

##### Sequences.

ITS from HMAS 254613 (MT676394).

##### Distribution.

China, France.

#### Nectria nigrescens

Taxon classificationFungiValvatidaGoniasteridae

Cooke, Grevillea 7(no. 42): 50, 1878.

E8F37C42-2E12-5D21-A4AC-CD45C1308656

##### Specimen examined.

China, Gansu Province, Shandan, Yanzhishan, on rotten twigs of broadleaf tree, 25 August 2018, Z.Q. Zeng, H.D. Zheng & X.C. Wang 12085 (HMAS 255802).

##### Sequences.

ITS (MT676393) and LSU (MT669265).

##### Distribution.

Canada, China, France, Germany, United Kingdom, United States.

#### Thelonectria discophora

Taxon classificationFungiHypocrealesNectriaceae

(Mont.) P. Chaverri & C. Salgado sensu lato, in Chaverri, Salgado, Hirooka, Rossman & Samuels, Stud. Mycol. 68: 76, 2011.

042DDDA5-E4BF-5659-94DE-65DED8B51495

##### Specimen examined.

China, Gansu Province, Wuwei, Chashugou, on rotten twigs, 26 August 2018, Z.Q. Zeng, H.D. Zheng & X.C. Wang 12149 (HMAS 279710).

##### Distribution.

Chile, China, United Kingdom.

#### Thyronectria rosellinii

Taxon classificationFungiHypocrealesNectriaceae

(Carestia) Jaklitsch & Voglmayr, Persoonia 33: 204, 2014.

EFB6DD5C-9C87-54CC-A92B-C6665C5DE7C9

##### Specimens examined.

China, Gansu Province, Wuwei, Chashugou, on rotten twigs, 26 August 2018, Z.Q. Zeng, H.D. Zheng & X.C. Wang 12150, 12151, 12154 (HMAS 279711, 255821, 255822).

##### Distribution.

Canada, China, France, Germany, Italy, Japan, United States.

#### Thyronectria zangii

Taxon classificationFungiHypocrealesNectriaceae

(Z.Q. Zeng & W.Y. Zhuang) Voglmayr & Jaklitsch, in Voglmayr, Akulov & Jaklitsch, Mycol. Progr. 15: 934, 2016.

72CF91E0-C30F-5364-B3C4-86B1179868DD

##### Specimens examined.

China, Gansu Province, Wuwei, Chengshanqizu, on rotten twigs of *Populus* sp., 28 August 2018, Z.Q. Zeng, H.D. Zheng & X.C. Wang 12285, 12286 (HMAS 255823, 255824).

##### Sequences.

ITS from HMAS 255824 (MT676392).

##### Distribution.

China.

#### Trichoderma alutaceum

Taxon classificationFungiHypocrealesHypocreaceae

Jaklitsch, Fungal Diversity 48: 69, 2011.

5F64631D-AD2B-5266-9D8E-C8B50DBAB40B

##### Specimen examined.

China, Gansu Province, Zhangye, Dayekou, on mossy ground under *Picea asperata*, 3 September 1958, Q.M. Ma 890 (HMAS 23955).

##### Distribution.

Austria, China, Denmark, Finland, Germany, Japan, Sweden, United Kingdom, United States.

#### Trichoderma paraviridescens

Taxon classificationFungiHypocrealesHypocreaceae

Jaklitsch, Samuels & Voglmayr, Persoonia 31: 128, 2013.

AD651CFC-0E9C-5EA1-9842-2B3D49B5E976

##### Specimen examined.

China, Gansu Province, Tianzhu, Haxi, on rotten twigs, 28 August 2018, Z.Q. Zeng, H.D. Zheng & X.C. Wang 12282 (HMAS 255825).

##### Sequences.

ITS (MT676391) and TEF1 (MT674562).

##### Distribution.

Austria, China, Colombia, France, Germany, Greece, Iran, Italy, Japan, Mexico, Spain, Switzerland, United States.

#### Trichoderma virens

Taxon classificationFungiHypocrealesHypocreaceae

(J.H. Mill., Giddens & A.A. Foster) Arx, Beih. Nova Hedwigia 87: 288, 1987.

2B845D11-2A73-5AD6-BF6A-ACDDC8A1B1EE

##### Specimen examined.

China, Gansu Province, Yongchang, in soil, April 2016, K. Chen TC896.

##### Distribution.

China, United States.

## Discussion

The genus *Stylonectria*, typified by *S. applanata*, was established as a monotypic genus by Höhnel (1915). It was included in *Nectria* by Booth (1959) and then treated as a synonym of *Cosmospora* Rabenh. by Rossman et al. (1999). Gräfenhan et al. (2011) resurrected the generic name *Stylonectria* and accepted four species. Lechat et al. (2015) described *S. norvegica* Lechat, J. Fourn. & Nordén from Norway. The morphological features of *S. qilianshanensis*, such as red perithecia growing on other ascomycetes, with flattened discoid apices, and two-layered wall bearing thick-walled outer cells, fit well the generic concept defined by Gräfenhan et al. (2011), which was confirmed by sequence analyses of the ACL1, ITS, and RPB2 regions. *Stylonectria qilianshanensis* is associated with *S. wegeliniana* (BIPP/MPBP = 100%/76%). *Stylonectria purtonii* [as *Cosmospora purtonii* (Grev.) Rossman & Samuels] on carbonaceous pyrenomycetes was the only species of the genus reported from China (Zhang and Zhuang 2003).

For some nectriaceous fungi, substrate type was considered of taxonomic importance (Zeng and Zhuang 2016). Species of *Stylonectria* are fungicolous and sometimes host-specific. For example, *S. applanata* is known only from *Melogramma bulliardii* Tul. & C. Tul. on *Corylus avellana*, *S. carpini* is restricted to a pyrenomycete on *Carpinus*, and *S. wegeliniana* colonizes solely on *Hapalocystis bicaudata* Fuckel on *Ulmus glabra* (Gräfenhan et al. 2011; Lechat et al. 2015). Similarly, *S. qilianshanensis* occurs on decayed fruit bodies of an ascomycete on the bark of *Picea asperata*. However, *S. norvegica* and *S. purtonii* have slightly wider host ranges. The former occurs on pyrenomycetes on Betulaceae and Fagaceae, while the latter is found on pyrenomycetes on coniferous trees. *Stylonectria* is currently a poorly known genus. Further investigations may provide useful information about its selection of hosts or substrates.

Gross morphology like stroma, ascospore features and asexual states were stressed for generic delimitations of the hypocrealean fungi. Species of Hypocreaceae possessing clavate to cylindrical, fleshy, bright-colored stromata were previously accommodated in *Podostroma* P. Karst. (Karsten 1892; Rossman et al. 1999). The accumulated molecular evidence argued that shape of fruit body is not phylogenetically distinctive within genus. Chamberlain et al. (2004) then synonymized *Podostroma* with *Hypocrea* Fr. (= *Trichoderma*). The diagnostic characteristics for *Trichoderma* include substrate, fruit body gross morphology, anatomy, extent of fertile region, surface pigmentation of stromata, and ascospore shape, size and ornamentation (Chamberlain et al. 2004). The genera *Aphysiostroma* Barrasa, A.T. Martinez & G. Moreno, *Pseudohypocrea* Yoshim. Doi, and *Sarawakus* Lloyd, which possess gliocladium-, trichoderma- and verticillim-like asexual states, were also synonymized with *Trichoderma* (Jaklitsch et al. 2014; Jaklitsch and Voglmayr 2015; Zeng and Zhuang 2017). The taxonomic position of *T. gansuanum* is confirmed by the combined sequence analyses of RPB2 and TEF1 regions and morphological characteristics, such as the stipitate, clavate, upright stromata, cylindrical asci, and disarticulating ascospores. A few stipitate species of *Trichoderma* have not been cultured or linked to asexual states (Chamberlain et al. 2004). Knowing the whole fungus is surely our future goal.

## Supplementary Material

XML Treatment for Stylonectria qilianshanensis

XML Treatment for Trichoderma gansuanum

XML Treatment for Hypomyces cervinigenus

XML Treatment for Hypomyces chrysospermus

XML Treatment for Hypomyces lateritius

XML Treatment for Hypomyces perniciosus

XML Treatment for Hypomyces rosellus

XML Treatment for Hypomyces stephanomatis

XML Treatment for Hypomyces tremellicola

XML Treatment for Nectria asiatica

XML Treatment for Nectria berberidicola

XML Treatment for Nectria nigrescens

XML Treatment for Thelonectria discophora

XML Treatment for Thyronectria rosellinii

XML Treatment for Thyronectria zangii

XML Treatment for Trichoderma alutaceum

XML Treatment for Trichoderma paraviridescens

XML Treatment for Trichoderma virens
